# 

**Published:** 1885-08

**Authors:** 


AUGUST. 1885.

### THE AMERICAN JOURNAL OF DENTAL SCIENCE.

PUBLISHED MONTHLY.

$2.50 PER ANNUM.

EDITED BY

F. J. S. GORGAS, M. D., D. D. S.

Vol. 19. THIRD SERIES. No 4.

BALTIMORE:
SNOWDEN & COWMAN.

LONDON:

TRUBNER & CO., 60 PATERNOSTER ROW.

1883.

PAYABLE IN ADVANCE.

